# "NO TIME TO OVERTHINK IT, JUST DO IT'': AREA AGENCIES ON AGING AND INCLUSIVE COMMUNITY ENGAGEMENT

**DOI:** 10.1093/geroni/igac059.294

**Published:** 2022-12-20

**Authors:** Traci Wilson, Elizabeth Blair

**Affiliations:** USAging, Washington, District of Columbia, United States; USAging, Washington, District of Columbia, United States

## Abstract

AAAs have always been deeply engaged in addressing the health-related social needs of older adults from historically marginalized and underserved communities. As a confluence of COVID and social injustices moved issues of racial equity and inclusion to the forefront of national conversation, AAAs refocused their efforts to identify and address health inequities related to the social determinants of health (SDOH). One result of the COVID-19 pandemic and the urgency to reach and vaccinate older adults was that AAAs quickly formed new partnerships with culturally specific organizations. These relationships have developed and resulted in expanding culturally responsive service delivery. Presenters will describe this and other findings from a mixed methods study about AAA initiatives that seek to improve access and equity, such as inclusive community needs assessments, equity analyses that leverage census data to identify areas of greatest SDOH disparities, and dashboards to compare service recipients with community demographics.

